# Persistent Urinary Podocyte Loss following Preeclampsia May Reflect Subclinical Renal Injury

**DOI:** 10.1371/journal.pone.0092693

**Published:** 2014-03-24

**Authors:** Wendy M. White, Angelica T. Garrett, Iasmina M. Craici, Steven J. Wagner, Patrick D. Fitz-Gibbon, Kim A. Butters, Brian C. Brost, Carl H. Rose, Joseph P. Grande, Vesna D. Garovic

**Affiliations:** 1 Department of Obstetrics and Gynecology, Mayo Clinic, Rochester, Minnesota, United States of America; 2 Division of Nephrology and Hypertension, Mayo Clinic, Rochester, Minnesota, United States of America; 3 Division of Biomedical Statistics and Informatics, Mayo Clinic, Rochester, Minnesota, United States of America; 4 Department of Laboratory Medicine and Pathology, Mayo Clinic, Rochester, Minnesota, United States of America; National Center for Scientific Research Demokritos, Greece

## Abstract

**Objective:**

Studies have shown that podocyturia, i.e., urinary loss of viable podocytes (glomerular epithelial cells), is associated with proteinuria in preeclampsia. We postulated that urinary podocyte loss may persist after preeclamptic pregnancies, thus resulting in renal injury. This may lead to future chronic renal injury. In addition, we compared the postpartum levels of the angiogenic factors, which previously have been associated with preeclampsia, between normotensive versus preeclamptic pregnancies.

**Study Design:**

The diagnosis of preeclampsia was confirmed using standard clinical criteria. Random blood and urine samples were obtained within 24 hours prior to delivery and 5 to 8 weeks postpartum. Urine sediments were cultured for 24 hours to select for viable cells and staining for podocin was used to identify podocytes. Serum samples were analyzed for the levels of angiogenic markers using ELISA (enzyme-linked immunosorbent assay) methodology.

**Results:**

At delivery, preeclamptic patients (n = 10) had significantly higher proteinuria (p = 0.006) and podocyturia (p<0.001) than normotensive pregnant patients (n = 18). Postpartum proteinuria was similar between these two groups (p = 0.37), while podocyturia was present in 3 of 10 women with preeclampsia and in none of the normotensive controls (p = 0.037). Angiogenic marker levels, including placental growth factor, soluble vascular endothelial growth factor receptor fms-like tyrosine kinase receptor-1 and endoglin, were not significantly different between women with preeclampsia and women with a normotensive pregnancy, either at delivery or postpartum.

**Conclusion:**

Persistent urinary podocyte loss after preeclamptic pregnancies may constitute a marker of ongoing, subclinical renal injury.

## Background

Preeclampsia is a pregnancy-specific disorder clinically characterized by hypertension and proteinuria that occurs after 20 weeks of gestation [1]. Affecting 6% of pregnancies, it remains one of the leading causes of both maternal and fetal morbidity and mortality worldwide. There is increasing evidence that preeclampsia is an under-recognized risk factor for cardiovascular disease [2]. In addition, evidence has emerged indicating that the affected women may be at risk for adverse renal outcomes later in life. Several cohort studies have suggested that the future risk of microalbuminuria is increased in those with a history of preeclampsia [3], [4]. Microalbuminuria is an important marker of preclinical atherosclerosis that is associated with cardiovascular mortality in postmenopausal women [5], along with the progression of chronic kidney disease [6]. In addition, women with histories of preeclamptic pregnancies are at risk for end-stage renal disease (ESRD) later in life [7], [8]. However, the underlying mechanisms of the association between preeclampsia and future microalbuminuria/ESRD have not been studied.

To-date, several studies have demonstrated that proteinuria in preeclampsia is associated with podocyturia, i.e., urinary excretion and loss of glomerular epithelial cells, also known as podocytes [9]–[16]. Podocytes are terminally differentiated cells that are situated on the outer aspect of the glomerulus, a highly-specialized functional unit of the kidney with selective permeability that allows for the free passage of water and solutes, but not protein. Foot processes of the neighboring podocytes interdigitate and connect via specialized cell-to-cell junctions, also known as glomerular slit diaphragms, and comprise the main size-selective filtration barrier in the kidney. The role of podocytes in preventing leakage of protein into the urine, as well as their dysregulation in proteinuric renal diseases, is well-documented [17]. As podocytes are terminally differentiated cells, they cannot divide. Consequently, podocyte loss from the glomerulus may lead to a disruption of the glomerular filtration barrier, thus preceding overt proteinuria [18], [19]. Our recent study showed that podocyturia predates the clinical features of preeclampsia, suggesting that podocyte loss may be mechanistically related to subsequent proteinuria [20].

The first aim of this study was to examine whether podocyturia, a marker of glomerular injury, persists after delivery. We postulate that ongoing urinary podocyte loss postpartum may contribute to chronic kidney injury, which has been documented years after the affected pregnancies [4], [7], [8]. In addition, previous studies have indicated that the degree of podocyturia at the time of delivery correlates with levels of placental growth factor (PlGF) [20], which, along with other angiogenic factors, such as soluble vascular endothelial growth factor (VEGF) receptor fms-like tyrosine kinase receptor-1 (sFlt-1) and endoglin, previously have been implicated in the pathogenesis of preeclampsia [21], [22]. In the second aim, we sought to compare the postpartum levels of the angiogenic factors between normotensive versus preeclamptic pregnancies.

## Materials and Methods

### Study Participants

This study was approved by the Mayo Clinic Institutional Review Board. Written consent was obtained from all women prior to enrollment in the study. The diagnosis of preeclampsia was established based on the presence of the following criteria [1]: a) new-onset hypertension after 20 weeks of gestation, defined as a blood pressure of ≥140/90 mm Hg, b) proteinuria, defined as ≥300 mg of protein in a 24-hour urine specimen, and/or 1+ (30 mg/L) proteinuria on dipstick urinalysis, in the absence of a urinary tract infection We also included women with severe forms of preeclampsia, such as eclampsia and HELLP (hemolysis, elevated liver enzymes, low platelets) syndrome, the diagnoses of which were confirmed on the basis of previously published criteria [23]. Our control group was composed of healthy, normotensive pregnant women without hypertension and proteinuria at any time during the pregnancy or at delivery.

Our initial cohort consisted of 273 women who were enrolled during their first prenatal visits, and then followed prospectively during their pregnancies. Pregnancy outcomes were ascertained at the time of hospitalization for delivery. Fifteen women developed preeclampsia, two of whom demonstrated additional characteristic findings of HELLP syndrome; 15 developed gestational hypertension and 204 had normotensive pregnancies. Six women had uncomplicated twin pregnancies and 33 women had one or more of the following pregnancy complications other than preeclampsia and gestational hypertension: gestational diabetes (n = 16), intrauterine growth retardation (n = 5), and pre-term delivery (n = 17). Our study cohort consisted of a total of 28 patients, 10 patients with preeclampsia/HELLP and 18 normotensive controls for whom blood and random urine samples were available both within 24-hours prior to delivery and at 5–8 weeks postpartum.

### Sample Processing and Analysis

**Figure 1:**
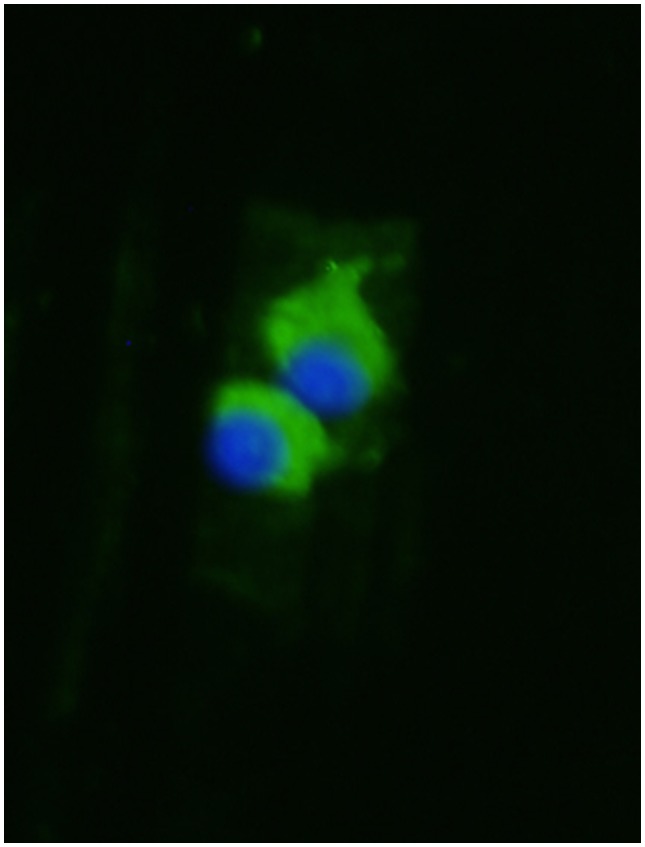
Podocyturia assay. Hoechst nuclear staining (blue); podocin antibody followed by a secondary fluorescein isothiocyanate-labeled antibody (green).

Serum samples were analyzed for the levels of angiogenic markers (sFlt-1, PlGF, and endoglin) using Quantikine ELISA (enzyme-linked immunosorbent assay) kits (R&D Systems, Minneapolis, MN). Aliquots of the midstream urine samples (50–100 mL) were collected in sterile containers and processed as previously described [9]. Briefly, urine albumin, total protein, and creatinine concentrations were measured using standard methods on a Hitachi 911 Chemistry Analyzer (Roche Diagnostics, Indianapolis, IN) and a predicted 24-hour urine protein was estimated from the protein/creatinine (P/Cr) ratio. Urinary sediments from the respective specimens were used for podocyte studies. Podocytes were identified by staining with a podocin antibody (1∶200, Sigma) after culturing the urinary sediments for 24 hours as previously described [9]. Nucleated, positive-staining cells were determined to be podocytes (Figure 1).

### Statistical Methods

The amount of 24-hour proteinuria was estimated from a P/Cr ratio and was expressed in gram protein/gram Cr. Podocyturia was expressed as a ratio of the number of podocytes per visual field to the milligrams of Cr of the respective urine sample. All continuous laboratory measurements were reported as median+interquartile range (IQR), 25^th^, 75^th^ percentile. Rank of values between groups (normotensive and preeclamptic pregnancies), at delivery and postpartum, were each compared using the Kruskal-Wallis rank sum test. Variables with normal distribution were expressed as mean ± standard deviation (SD), and compared between the groups using a two sample t-test. Counts of the number of subjects positive for podocyturia between the preeclampsia and normotensive groups, at either delivery or postpartum, were compared using Fisher’s exact test.

## Results

Urine and plasma samples within 24 hours of delivery and 5–8 weeks postpartum were available for 10 patients with preeclampsia/HELLP and 18 normotensive controls. The women with normotensive pregnancies did not differ significantly from those with preeclampsia/HELLP with respect to demographic parameters, such as age, number of gestations, parity, or nulliparous status (Table 1).

**Table 1:** Subject characteristics according to pregnancy outcome.

Variable	Normotensive (n = 18)	PE/HELLP (n = 10)	*P* Value
Maternal Age, years; mean (±SD)	27.4 (2.5)	28.7 (5.9)	0.42
Gestations, n (%)			0.21
1	16 (89)	7 (70)	
2	1 (6)	2 (20)	
3	1 (6)	1 (10)	
Parity, n (%)			0.21
0	16 (89)	7 (70)	
1	1 (6)	2 (20)	
2	1 (6)	1 (10)	
Nulliparous, n (%)	16 (89)	7 (70)	0.21
Delivery Systolic, mean (±SD)	122.3 (6.2)	147.4 (6.6)	<0.001
Delivery Diastolic, mean (±SD)	72.5 (6.5)	92.0 (5.6)	<0.001
Gestational Age at delivery in days, mean (±SD)	279 (6)	272 (13)	0.06
Postpartum Systolic, mean (±SD)	105.6 (12.0)	105.8 (13.5)	0.96
Postpartum Diastolic, mean (±SD)	64.4 (8.4)	70.8 (9.2)	0.08
Follow-up in years, mean (±SD)	4.1 (1.6)	3.9 (1.7)	0.77

T-test for continuous variables, Fisher’s exact test for discrete variables (for those with >2 levels, the lowest was compared to non-lowest as a group).

At the time of delivery, women with preeclampsia/HELLP had significantly higher systolic blood pressures (147.4±6.6 mm Hg versus 122.3±6.2 mm Hg, p<0.001), diastolic blood pressures (92.0±5.6 mm Hg versus 72.5±6.5 mm Hg, p<0.001) (Table 1), and P/Cr ratio (median 0.61 g/g Cr; IQR 0.23, 1.14 versus 0.08 g/g Cr; IQR 0.07, 0.17) compared to the normotensive controls (Table 2). Podocyturia was present in all patients with preeclampsia/HELLP (median 0.92 podocytes/mg creatinine; IQR 0.44, 1.37) and in only one patient (0.06 podocytes/mg creatinine) from the control group (p<0.001) (Table 2).

**Table 2:** Clinical Parameters and Angiogenic Markers at 2: Delivery and Post-Partum.

Variable	Time Point	Normotensive (n = 18)		PE (n = 10)		P Value^a^
		Median	IQR, 25^th^;75^th^ percentiles	Median	IQR, 25^th^;75^th^ percentiles	
Endoglin, ng/mL	Delivery	14.64	9.98;21.26	22.23	11.57;33.39	0.31
	Post-Partum	4.66	4.20;6.35	4.72	4.01;5.33	0.46
PlGF, pg/mL	Delivery	156.88	79.24;208.70	129.68	100.67;181.39	0.92
	Post-Partum	6.16	3.75;11.39	6.00	5.21;13.69	0.70
sFlt-1, pg/mL	Delivery	7352.42	4363.13;11918.70	7592.30	4996.00;13530.80	0.60
	Post-Partum	138.32	74.05;198.40	115.40	1.00;203.60	0.60
Protein/creatinine ratio, g	Delivery	0.08	0.07;0.17	0.61	0.23;1.14	**0.006**
	Post-Partum	0.03	0.01;0.04	0.04	0.03;0.04	0.37
Podocytes/creatinine ratio, mg	Delivery	0.00	0.00; 0.00	0.92	0.44; 1.37	**<0.001**
	Post-Partum	0.00	0.00; 0.00	0.00	0.00; 0.13	0.07
Presence of Podocyturia, n(%)	Delivery	1(6)		10(100)		**<0.001** b
	Post-Partum	0(0)		3(30)		**0.037** b

a Kruskal Wallis rank sum test.

b Fisher’s exact test.

NOTE: **Bold** indicates statistical significance, i.e. p≤0.05.

Postpartum samples were obtained at 44±7 days and 43±4 days after delivery for cases and controls, respectively. Postpartum systolic and diastolic blood pressures were similar between the women with preeclampsia/HELLP, p = 0.96 and p = 0.08, respectively (Table 1). Also, the P/Cr ratio was no different, median 0.04 g/g Cr (IQR 0.03, 0.04) for women with preeclampsia versus median 0.03 g/g Cr (IQR 0.01, 0.04) for those with normotensive pregnancies, p = 0.37 (Table 2). Podocyturia was present in 3 of the 10 women with preeclampsia/HELLP (median 0.00; IQR 0.00, 0.13) and was absent in all of the normotensive controls, p = 0.037 (Table 2). Angiogenic marker levels were not significantly different between women with preeclampsia/HELLP and the normotensive pregnancy controls either at delivery or postpartum (Table 2).

Long-term follow-up was not significantly different between normotensive (mean 3.9 years) and preeclamptic pregnancies (mean 4.1 years, p = 0.77). Among the 3 women who demonstrated podocyturia postpartum, one went on to develop 2+ proteinuria (measured by the dipstick method) and hypertension; one was diagnosed with an autoimmune connective tissue disorder, but had no urinalysis/renal function follow up; and the third patient had no documented renal or other events. Among the women with normotensive pregnancies, none of them developed hypertension or renal disease for the duration of follow-up. Interestingly, the one woman classified as normotensive and with podocyturia at delivery had two diastolic blood pressures >90 mm Hg, five hours apart, but delivered before an official clinical diagnosis of preeclampsia could be made.

## Discussion

This pilot study demonstrates that podocyturia may persist postpartum in 30% of women with preeclampsia/HELLP, despite the resolution of proteinuria. We and others have reported previously that podocyturia is present in patients during the acute phase of preeclampsia and commonly is absent in normotensive controls [9]–[16], [20], [24]. We have also shown that podocyturia precedes both the clinical symptoms of preeclampsia and the presence of proteinuria by weeks, and may serve as a more sensitive and specific predictor of preeclampsia than angiogenic markers [20]. However, little is known about the recovery process of podocyte injury after preeclamptic pregnancies, although proteinuria commonly resolves by 12 weeks postpartum. A single study of time course alterations in podocyturia in 11 preeclamptic patients reported the presence of podocyturia 4 days postpartum, with resolution of podocyturia in all but one woman at 1 month after delivery [10]. Among the 45 normotensive controls, nine exhibited mild-to-moderate podocyturia, but without proteinuria, four days after delivery. It is important to note that this study used the cytospin technique which may lead to strong a background signal from protein debris, erythrocytes, and other cells that may be present [25], particularly around the time of delivery when urine samples may be contaminated with decidual and amniotic cells and proteins. In addition, podocytes in this study were identified by staining for podocalyxin [10]. Given the variety of different cell types found in urinary sediment after delivery, staining with podocalyxin, which can be expressed by cells other than podocytes, might have resulted in false-positive results. Despite these technical problems, this study demonstrated significant differences between preeclamptic women, both at delivery and 4 days postpartum. Our study, based on the overnight culturing of urinary sediments and subsequent staining for podocin, a podocyte-specific protein, indicates that podocyturia may persist postpartum. This ongoing podocyte loss may be a possible underlying mechanism that links preeclampsia and future renal disease, including ESRD, and should be the subject of the future research. Of note, angiogenic markers were not different between women with normotensive versus preeclamptic pregnancies at the time of delivery, likely due to the fact that preeclampsia developed in term pregnancies, as alterations in angiogenic markers are most prominent in early preeclampsia (<34 gestational weeks). Similarly, these angiogenic markers were not different between women with normotensive and preeclamptic pregnancies at their postpartum visits.

Viable urinary podocytes have been documented in a variety of glomerular diseases, and particularly, during the active phases of disease [26]–[28]. Culturing of urinary podocytes increases specificity by removing dead and nonspecific cells; thus selecting for viable cells, based on their abilities to attach to collagen-coated slides. As podocytes are terminally differentiated cells that do not replicate [17], a critical loss of viable cells may lead to permanent renal injury. This has been demonstrated in a mouse model of selective podocyte depletion using diphtheria toxin [29], in which a single episode of podocyte injury resulted in glomerular destabilization and persistent podocyte loss, suggesting that changes in the structure of the kidney at the level of the podocyte barrier continue following the resolution of an acute injury. Therefore, the association between preeclampsia and renal disease later in life, which commonly is attributed to shared risk factors, such as obesity, diabetes mellitus, and autoimmune connective tissue disorders, may be due to immediate and long-term podocyte loss and structural changes that might contribute to the future risk for renal disease. Podocyturia plays a major role in the pathogenesis of focal segmental glomerular sclerosis [30], which, in turn, is a dominant finding in renal biopsies of women with a history of preeclampsia and persistent proteinuria [31]. Taken together, these data suggest that women with preeclamptic pregnancies and persistent proteinuria postpartum may have an underlying lesion of focal segmental glomerular sclerosis and ongoing podocyte loss, which may contribute to their increased risks for future kidney disease, including ESRD.

The primary limiting factor of our study is the relatively small sample size. Nevertheless, we feel that podocyte injury and subsequent shedding in the urine: (1) is inextricably linked to preeclampsia, (2) confirms the diagnosis at the time of acute presentation with high sensitivity and specificity, and (3) may serve as a biomarker for future renal and cardiovascular risk in the postpartum period. The podocyte culturing technique is technically complex, lengthy, and requires a high level of training and expertise, thus making it unsuitable for large scale studies. Newly developed technologies, based on either mass spectrometry [16] or RT-PCR [12], offer standardized and reproducible techniques for the detection of podocyte products. These methods are operator-independent and highly reproducible, and may facilitate future large-scale studies of the time course alterations of podocyturia after preeclamptic pregnancies.
